# On Plant Detection of Intact Tomato Fruits Using Image Analysis and Machine Learning Methods

**DOI:** 10.3390/s140712191

**Published:** 2014-07-09

**Authors:** Kyosuke Yamamoto, Wei Guo, Yosuke Yoshioka, Seishi Ninomiya

**Affiliations:** 1 Graduate School of Agricultural and Life Sciences, The University of Tokyo, 1-1-1, Midori-cho, Nishitokyo, Tokyo 188-0002, Japan; E-Mails: 8244012644@mail.ecc.u-tokyo.ac.jp (K.Y.); guowei@isas.a.u-tokyo.ac.jp (W.G.); 2 Graduate School of Life and Environmental Sciences, University of Tsukuba, 1-1-1, Tennodai, Tsukuba, Ibaraki 305-8573, Japan; E-Mail: yoshioka.yosuke.fw@u.tsukuba.ac.jp

**Keywords:** image analysis, fruit detection, machine learning, young fruit, tomato

## Abstract

Fully automated yield estimation of intact fruits prior to harvesting provides various benefits to farmers. Until now, several studies have been conducted to estimate fruit yield using image-processing technologies. However, most of these techniques require thresholds for features such as color, shape and size. In addition, their performance strongly depends on the thresholds used, although optimal thresholds tend to vary with images. Furthermore, most of these techniques have attempted to detect only mature and immature fruits, although the number of young fruits is more important for the prediction of long-term fluctuations in yield. In this study, we aimed to develop a method to accurately detect individual intact tomato fruits including mature, immature and young fruits on a plant using a conventional RGB digital camera in conjunction with machine learning approaches. The developed method did not require an adjustment of threshold values for fruit detection from each image because image segmentation was conducted based on classification models generated in accordance with the color, shape, texture and size of the images. The results of fruit detection in the test images showed that the developed method achieved a recall of 0.80, while the precision was 0.88. The recall values of mature, immature and young fruits were 1.00, 0.80 and 0.78, respectively.

## 1. Introduction

Fully automated yield estimation of intact fruits before harvesting is an important task in the field of precision agriculture. In fact, the procedure provides various benefits to farmers. For instance, performing site-specific management based on yield mapping reduces labor costs for cultivation management and harvesting, and optimizes the amount of materials required such as fertilizers and agricultural chemicals. Furthermore, yield estimation provides other benefits to post harvesting such as estimating the storage capacity required to store harvested fruits [1].

Until now, several studies have been conducted to estimate fruit yield. Many of these studies employed image-processing technologies to estimate the yields of fruits such as apples [1–9], citrus fruits [10–12], peaches [13,14], grapes [15,16] and mangoes [17,18]. These were employed because image-processing technologies can conduct object detection and counting accurately, quickly and non-destructively. In recent studies, Wang *et al.* [1] developed an image-processing crop yield estimation method for apple orchards. They developed two algorithms to detect both red and green apples from images. For detection of red apples, they conducted thresholding in a hue saturation value (HSV) color space. With regard to green apple detection, they evaluated the circularity of specular reflection on the apples. Zhou *et al.* [9] used only color features to detect and count apples in an image. They calculated the differences among red, green and blue values in RGB color space, and then set thresholds on these values to segment an image into fruit and background regions. Kurtulmus *et al.* [12] utilized an image-processing method for human face detection as a means of detecting citrus fruits. As a result, they could detect fruits under difficult situations such as occlusions, immature fruits and natural lighting conditions.

Although previous studies have developed numerous approaches to detect and count the fruits in an image, most of them required thresholds for features such as color, shape and size [5,9,17,18]. In addition, their performance strongly depended on the thresholds chosen, although optimal thresholds tended to vary with images. Furthermore, most of these methods have attempted to detect mature and immature fruits, although the number of young fruits is more important for prediction of long-term fluctuations in yield.

On the other hand, some researchers have used hyperspectral [19,20], thermal [4,21] and ultrasonic [22] imaging technologies for fruit detection. These technologies typically provide better results than conventional RGB color images for fruit detection. This is because objects with similar color may exhibit different reflectance in non-visible regions, and thereby, they can be easily distinguished. However, these technologies require expensive instruments compared with RGB cameras, and thus are not suitable for practical use. Therefore, it is desirable to develop a method that provides similarly accurate results using low cost RGB cameras.

In this study, we aimed to develop a method to accurately detect individual intact tomato fruits including mature, immature and young fruits on plant using a conventional RGB digital camera in conjunction with machine learning approaches, which have been being utilized in a wide range of biological studies [4,6,11,14,15,23–25]. The developed method was based on an image segmentation process involving three steps: pixel-based segmentation, blob-based segmentation and individual fruit detection. For each step, classification models were generated using the color, shape, texture and size of the images. The image segmentations were conducted using the generated models; therefore, the developed method does not require that threshold values be adjusted to each image. Finally, we evaluated the fruit detection and accuracy rate to identify the effectiveness of the developed method for yield estimation of intact tomato fruits.

## 2. Materials and Methods

### 2.1. Image Acquisition

Tomato plant images were acquired at the Tsukuba plant factory of the Institute of Vegetable and Tea Science (Ibaraki, Japan). For image acquisition, a digital single-lens reflex camera (EOS Kiss X3, Canon Inc., Tokyo, Japan) was set up in the plant factory. Images were acquired at 2-hour intervals during the nighttime from 5 February to 7 March 2013 for a total of 154 images. When capturing the images, the built-in flash of the camera was used as a light source. Fifty-four images were randomly selected from the 154 images, and 13 of the images were used for training, whereas the remaining images were used for testing.

### 2.2. Pixel-Based Segmentation

**Figure 1.**
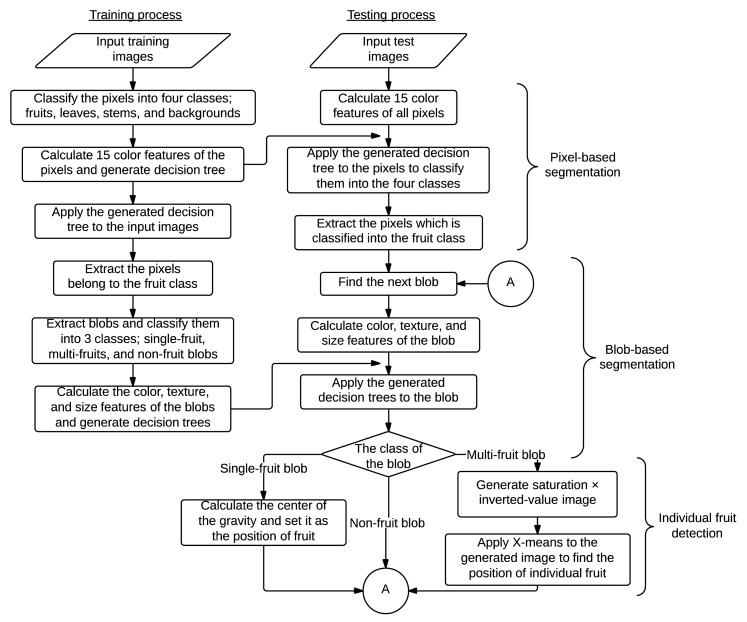
Flowchart of the developed method.

The developed method for fruit detection consists of three processes. A flowchart of the developed method is shown in Figure 1. The first process is pixel-based segmentation, which relies on a decision-tree-based segmentation model (DTSM) [26].

First, we manually labeled pixels of the training images into four classes (fruits, leaves, stems and backgrounds). We acquired a total of 140,000 pixels for the fruit class and 100,000 pixels for the leaf, stem and background classes, respectively, as the training dataset. We then extracted 15 color features (r, g, b; H, S, V; L*, a*, b*; L*, u*, v*; Y, Cb, Cr) defined in five ordinarily used color spaces (rgb, HSV, L*a*b*, L*u*v* and YCbCr) of the pixels. For the training dataset acquisition, we used a GUI application developed for the present research, with which the color features of the selected pixel and its 8-neighbor pixels are automatically extracted with a selected label such as “fruit”, “leaf”, “stem” and “background”. Color space transformation from RGB to the others was conducted using the function for color conversion in OpenCV 2.4.9 [27]. We built a decision tree based on the classification and regression tree (CART) classifier [28]. The generated decision tree was applied to the test images to classify their pixels into the chosen classes.

### 2.3. Blob-Based Segmentation

As pixel-based segmentation is not perfect, numerous misclassifications were observed in the results of the process. Therefore, we conducted blob-based segmentation to remove such misclassifications.

**Figure 2.**
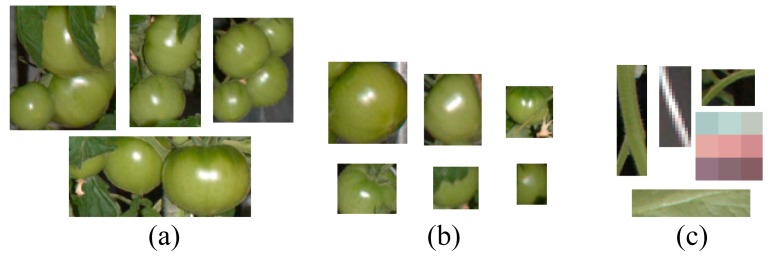
Examples of the training dataset for blob-based segmentation. (**a**) Multi-fruit blobs; (**b**) Single-fruit blobs; (**c**) Non-fruit blobs.

First, we extracted the pixels classified as the fruit class by pixel-based segmentation. Next, we calculated a minimal rectangle enclosing each connected region of the extracted pixels, and images were cropped with the calculated rectangles. In this study, these cropped areas are referred to as “blobs.” As misclassifications in the pixel-based segmentation process resulted in blobs that also included non-fruit regions, we conducted classification of the blobs using the random forest classifier [29]. Blobs obtained from the training images were manually classified into three classes: multi-fruit, single-fruit and non-fruit blobs (shown in Figure 2). We acquired a total of 30, 200 and 2000 blobs for multi-fruit, single-fruit and non-fruit blobs, respectively, as the training dataset. Then, we calculated the features of color, shape, texture and size of the classified blobs, and built decision trees using these features. For the color feature of the blobs, the average values of the 15 color features used in the pixel-based segmentation step were calculated. For the texture features, we converted the blobs to grayscale images and generated a histogram and gray level co-occurrence matrix (GLCM) [30]. Then, the contrast features of the histogram and the angular second moment of the GLCM, which, from our preliminary analysis, were considered important texture features enabling discrimination of fruit and non-fruit blobs, were calculated. For the size and shape features, the blob size measured by width × height and the ratio of width to height were used, respectively. The generated decision trees were applied to the blobs extracted from the test images to identify the blobs that contain one or more fruits.

We divided the blobs including fruits into two classes: single-fruit and multi-fruit blobs. The reason for doing so can be explained as follows. Because a tomato plant makes fruit clusters, some of the fruits were connected to other fruits as a result of pixel-based segmentation. For multi-fruit blobs, the position of individual fruit within the blob had to be estimated by an additional procedure, which will be explained in the next section, because our objective was to detect individual fruit in an image. On the other hand, with regard to single-fruit blobs, the additional procedure was not necessary because blobs were constructed so as to position the fruit close to the center of gravity of the blob. Furthermore, application of an unnecessary procedure would decrease the overall accuracy of the method. Thus, we prepared the two classes of fruit blobs.

### 2.4. Individual Fruit Detection in Multi-Fruit Blobs

As a result of blob-based segmentation, blobs containing no tomato fruit are eliminated. The blobs identified as single-fruit blobs contain one fruit; therefore, the center of gravity of the single-fruit blob can be used as the fruit position. A multi-fruit blob contains more than two fruits, but the actual quantities and the positions of the individual fruits are unknown. Therefore, the quantities and the positions must be predicted to detect all fruits in an image.

**Figure 3.**
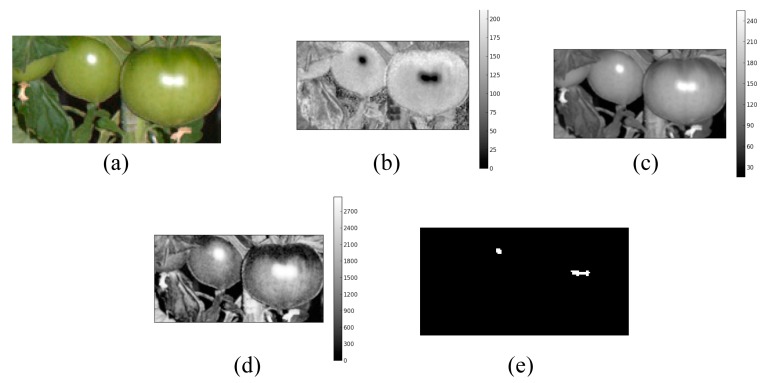
Example of detection of the overexposed regions in a multi-fruit blob. (**a**) Original image; (**b**) Saturation channel in the HSV image; (**c**) Value channel in the HSV image; (**d**) Image generated by multiplication of the value and inverted saturation channels; (**e**) Detected regions by applying *X*-means clustering.

Figure 3 illustrates the procedure of individual fruit detection in a multi-fruit blob. As shown in Figure 3a, there is an overexposed region caused by the camera flash on each fruit. We used this region to detect the individual fruit in a multi-fruit blob. First, the image of a multi-fruit blob was converted into HSV color space. It was observed that the overexposed regions in the HSV image exhibited low saturation and high value (Figures 3b and 3c; therefore, we inverted the saturation channel and multiplied it by the value channel in the HSV image. Figure 3d shows an image generated by this procedure where the overexposed regions exhibit fairly high values relative to the other regions. Thus, the overexposed regions can be easily extracted compared with the original saturation and value channels in the HSV image.

In the next step, we applied the *X*-means clustering algorithm [31] to the generated image to extract the overexposed region. *X*-means clustering operates on the basis of *K*-means clustering [32]. The main drawback of *K*-means clustering is that the number of clusters has to be determined by users, although the optimal *K* depends on datasets. On the other hand, in *X*-means clustering, the splitting decision of clusters is made based on the Bayesian information criterion of the clusters, thereby automatically determining the number of clusters. By applying the *X*-means algorithm, pixels in an image are classified into *X* clusters based on the values calculated by multiplication of the value and inverted saturation channels. Then, we calculated the average values for each cluster and extracted only the pixels that belong to the cluster with the highest average value. Finally, the center of gravity of each connected region within the extracted pixels was used as the position of an individual fruit in the multi-fruit blob.

### 2.5. Evaluation of the Developed Method

To evaluate the performance of the developed method of fruit detection, we calculated recall and precision:

(1) $$\text{Recall}=\frac{\text{Total number of detected fruits in images}}{\text{Total number of fruits in images}}$$

(2) $$\text{Precision}=\frac{\text{Total number of relevant detections in images}}{\text{Total number of detections in images}}$$

where recall is the measure of completeness, whereas precision is the measure of exactness.

**Figure 4.**
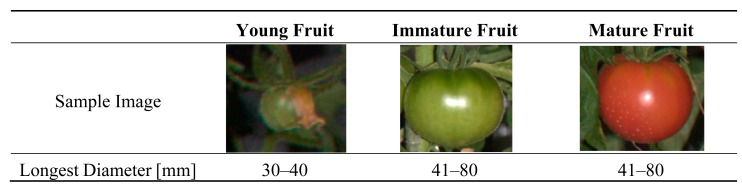
Definition of the growth stages of tomato fruit used in the present study. Tomato fruits were classified into three categories based on their color and size.

In this study, we divided the fruits into three growth stages based on their appearances. The definitions of the growth stages are described pictorially in Figure 4. We calculated the recall for fruits at each growth stage respectively, and recall and precision values for fruits at all growth stages.

## 3. Results and Discussion

### 3.1. Pixel-based Segmentation

**Figure 5.**
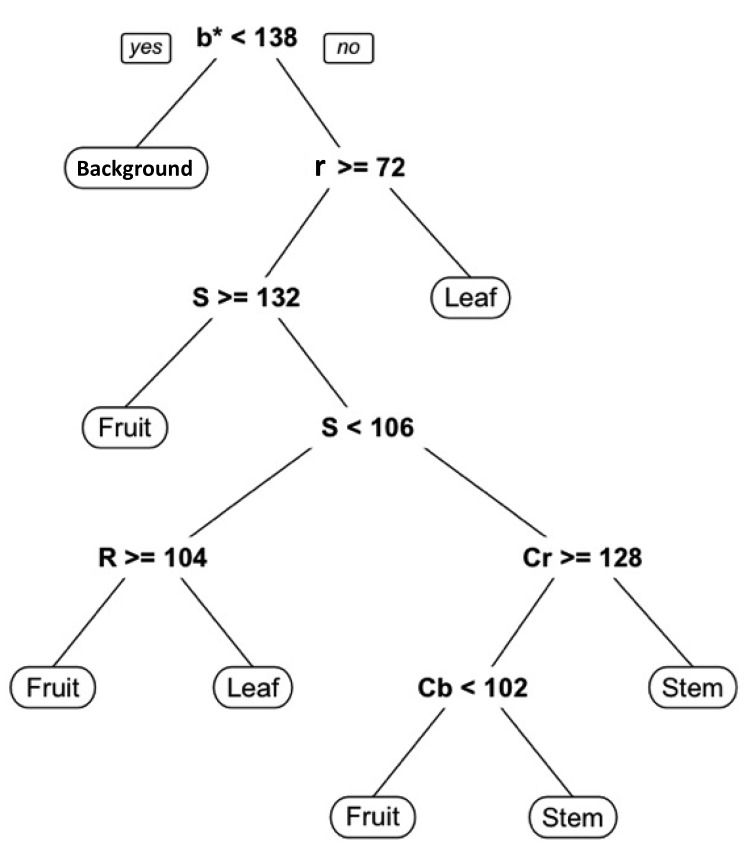
Generated decision tree for pixel-based segmentation.

**Figure 6.**
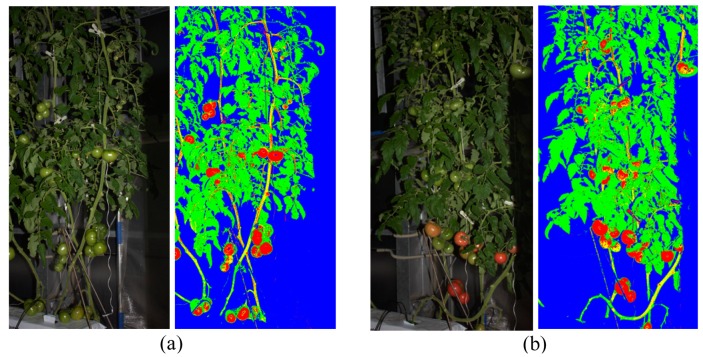
Examples of pixel-based segmentation. Left images show the original images. Right images show the result of the pixel-based segmentation. Pixels drawn with red, green, yellow, and blue colors are pixels classified into fruit, leaf, stem and background, respectively.

**Figure 7.**
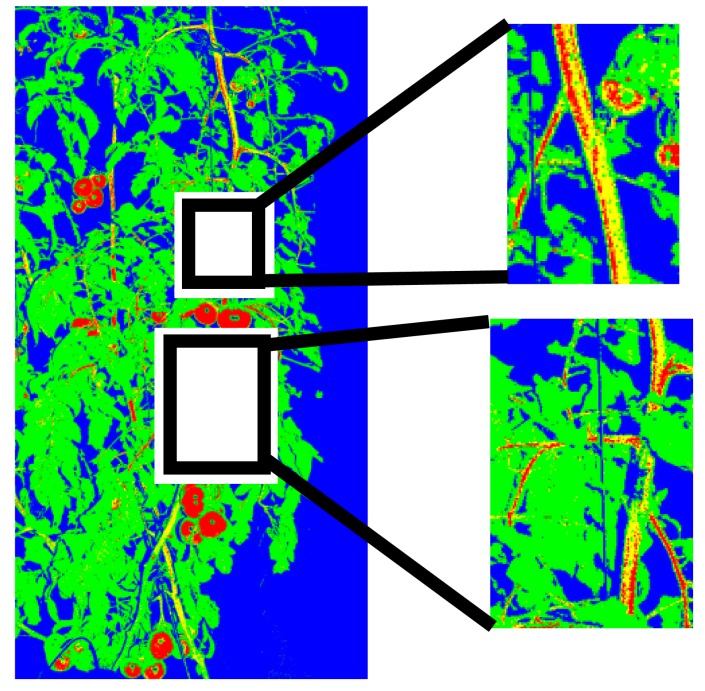
Examples of pixels misclassified by pixel-based segmentation. In particular, the stem parts tend to be misclassified as the fruit class.

Figure 5 presents the generated decision tree for pixel-based segmentation, and it shows that the color features b* of L*a*b*, r of RGB, S of HSV, and Cr and Cb of YCrCb were finally used. Figure 6 shows the results of pixel-based segmentation of the test images. Although some pixels, particularly those of stem parts, were misclassified as the fruit class, the test images taken at different stages of plant maturity were roughly segmented into the correct classes. Figure 7 provides examples of misclassification by pixel-based segmentation. Misclassification typically occurs when (i) stems and tomato fruits, particularly young fruits, and (ii) the highlight on fruits and objects such as pole in background have a similar color. However, performing blob-based segmentation reduces such misclassifications.

Application of pixel-based segmentation extracts pixels belonging not only to fruit but also the leaf and stem parts of the images. Therefore, other important information, such as the amount and condition of foliage, stem lengths and branching [25], could be measured in a future study.

### 3.2. False Positive Elimination Based on Blob Features

**Figure 8.**
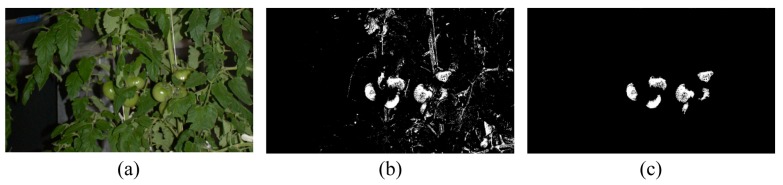
Example of false positive elimination by blob-based segmentation. (**a**) Original image (the middle section of one of the test images); (**b**) Result of pixel-based segmentation, where a pixel color of white belongs to the fruit class; (**c**) Result of false positive elimination by blob-based segmentation.

Blob-based segmentation could eliminate the false positives generated from pixel-based segmentation. Figure 8 shows an example of such elimination. In the case of Figure 8, 8715 blobs were observed in the result of pixel-based segmentation, although there are only seven fruits in the image. However, the number of candidate blobs was reduced to seven by performing blob-based segmentation, and as a result, seven fruits were detected. Thus, we could eliminate a great many false positives generated from the pixel-based segmentation process by performing blob-based segmentation.

**Figure 9.**
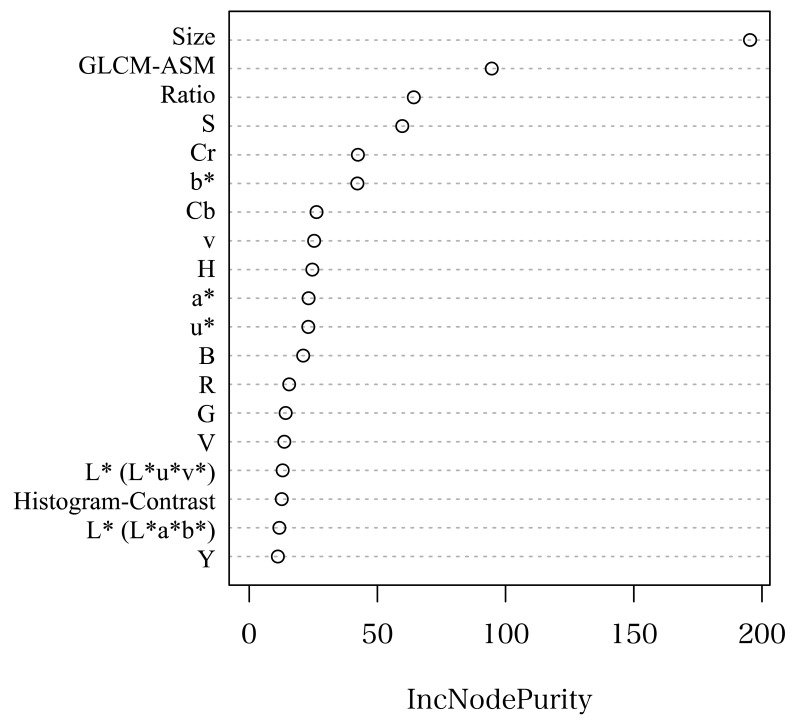
Importance of features based on the classification of blobs using the random forest classifier during blob-based segmentation. The horizontal axis shows the mean decrease Gini, which provides an indication of the importance of variables.

Figure 9 illustrates the features calculated during blob-based segmentation ordered by an importance measure. During blob-based segmentation, the blob size was the key feature for classifying the three types of blobs considered (*i.e.*, multi-fruit, single-fruit and non-fruit) because, with regard to the multi-fruit and the non-fruit blobs, most of the non-fruit blobs consisted of several pixels that may have been caused by noise, whereas the multi-fruit blobs consisted of many more pixels than the non-fruit blobs. With respect to the multi-fruit and single-fruit blobs, the multi-fruit blobs were typically larger than the single-fruit blobs because the former included more than two fruits. However, blob sizes may change with changing distance between the camera and the tomato plant. This was not a troublesome condition in this study because we used images always taken from same distances, and a sizing error was less than approximately 20% between the near and far sides of tomato plant. However, when blob-based segmentation is applied to images taken from various distances, some countermeasures are required such as providing a scale beside a tomato plant to determine its relative size. Furthermore, combining 3D information with color images can solve such problems. Color information and distance to an object can be simultaneously measured using consumer depth cameras such as Microsoft Kinect (Microsoft, Seattle, WA, USA) and ASUS Xtion (ASUS, Taipei City, Taiwan). Once the distance to an object is measured, the relative size of the object in the image can be determined. According to Menesatti *et al.* [33], the actual size and weight of an object can be estimated using stereovision. Since the stereovision system developed in their study can be used even under the field condition with high accuracy, it is expected to improve our method in the future study.

In the developed method, we cannot detect fruits when none of the relevant pixels are classified into the fruit class by pixel-based segmentation. In fact, some fruits, particularly young fruits, were classified into leaf and stem classes, and therefore went undetected. In a future study, we will consider not only false positive elimination but also false negative detection during blob-based segmentation to improve the performance of the developed method.

### 3.3. Fruit Detections

Table 1 describes the results of fruit detection from the test images. The developed method could detect 80% of all fruits in the test images. In addition, precision was 0.83; thus, the accuracy of the developed method was also very high. Furthermore, the recall value for young fruits was high (0.78), despite the fact that young fruits are very difficult to detect as their color is similar to that of the stem class and their size is quite small. With regard to immature and mature fruits, we could achieve high recall values of 0.81 and 1.00, respectively.

**Figure 10.**
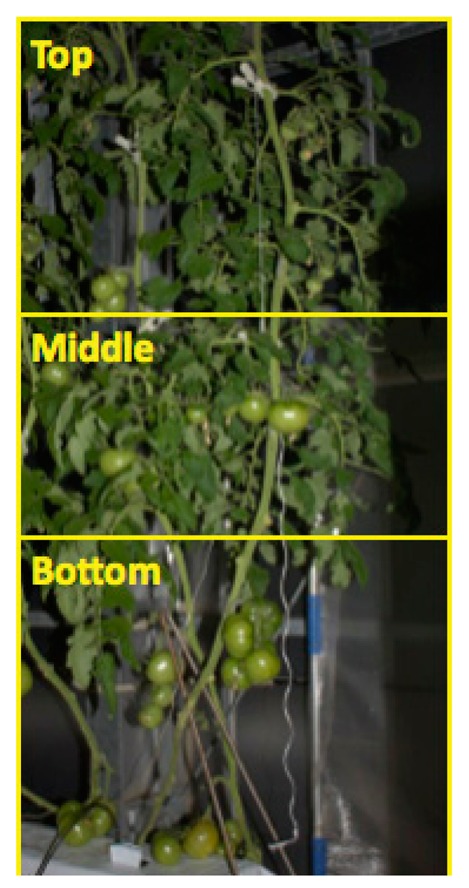
Example of image partition for the part-by-part fruit detection.

In this study, we also conducted part-by-part fruit detection: we divided the tomato images vertically into three parts, top, middle and bottom, as shown in Figure 10.

All images were cropped at the same coordinates, which were arbitrarily determined based on the distribution of growth stages of fruit in the test images. The top part includes immature and young fruits. The middle part is composed of only immature fruits. The bottom part includes both immature and mature fruits. We conducted model constructions and fruit detection on each part individually. The results of the part-by-part fruit detection are also shown in Table 1. Recall and precision were generally improved by applying the developed method to each part individually. This is owing to the fact that variations in the appearances of components, such as fruits, stems and leaves, became smaller by dividing the images vertically because the appearances of the components reflect differences in the plant's growth periods from bottom to top. Although division of the images improved the performance of the developed method, a method to automatically divide the images in accordance with the differences in the growth stages of a plant in an image is yet to be developed. Therefore, part-by-part fruit detection is still impracticable.

**Figure 11.**
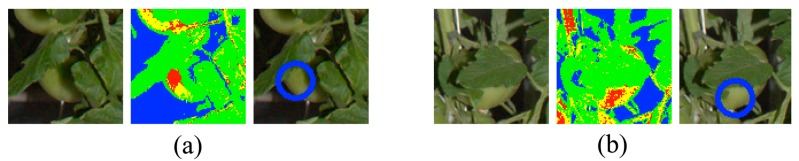
Examples of fruit detection under difficult conditions. The original images are to the left. The middle images show the results of pixel-based segmentation. The images to the right show the results of fruit detection.

The developed method could detect fruits under difficult conditions, such as occlusion, as shown in Figure 11, because we conducted pixel-based segmentation first. It is quite difficult for other methods, such as that based on sub-windows [12], to detect occluded fruits because occluding objects influence the features in the sub-window. To avoid this, an optimal sub-window size must be determined for each fruit. On the other hand, by our method, the result of pixel-based segmentation determines the blob size, which is the smallest rectangle enclosing connected fruit pixels; thereby, the effects of other objects are minimized.

In this study, we focused on the detection of tomato fruits in an image. However, the number of fruits obtained from 2D images is not always the same as that on the actual plant because some fruits may be completely occluded by leaves, stems, and even other fruits, and hence, cannot be detected. To solve this problem, we have to conduct statistical analyses of the results of our approach in conjunction with the ground truth. For instance, we obtained a regression equation by performing regression analysis; thus, we can estimate the actual number of fruits from our results. Relative amount of leaves that can be estimated from pixel-based segmentation may be useful to improve the regression as the information to estimate the number of occluded fruits. Using 3D point clouds for the detection of fruits is also expected to improve the effect of occlusions. Nowadays, several methods have been developed to easily construct a 3D point cloud from 2D images taken from multiple viewpoints without any calibrations [34–36]. We can easily construct 3D point cloud of plants with conventional digital cameras by using such methods. In particular, Dey *et al.* [25] utilized such a method to extract grapes from a 3D point cloud. Although the researchers did not discuss the advantages of using a 3D point cloud with regard to their results, the fruits were detected with high accuracy.

In this study, we used the images of the same tomato plants taken with a fixed-point camera. However, it is necessary to analyze the images of all plants in a farm field to generate more useful information for farmers. The Tsukuba plant factory, where the tomato images used in this study were taken, has the equipment to incorporate an autonomous travel robot into the process. In a future study, we plan to mount a camera on the robot to acquire images of all tomato plants in the farm field, and to generate the yield map by analyzing them.

### 3.4. Developed Method

The developed method in this study consists of three steps: pixel-based segmentation, blob-based segmentation and individual fruit detection. In the first two steps, decision trees were generated based on features, such as color, shape, texture and size, and image segmentations were conducted using the generated trees. In addition, we used *X*-means clustering to automatically determine the optimal number of clusters and to detect individual fruit in a multi-fruit blob. Accordingly, the developed method does not require an adjustment of the threshold values of each image for fruit detection. Most methods developed in related studies require such threshold adjustments that may vary from image to image, and which must be determined by users. On the other hand, the developed method can perform fully automated fruit detection once the training images are provided.

In this study, we used four types of features: color, shape, size and texture. Color features change depending on the position of fruits in a canopy, because lighting condition was not uniform completely. Hashimoto *et al.* [37] and Menesatti *et al.* [38] have conducted color calibration of images taken under the in-field condition. In the future study, these advanced technologies should be combined in our current method to make the color features invariant. For the shape feature, we used very simple one: the ratio of width to height of blob. A lot of sophisticated methods for shape analysis have been reported in recent studies [39,40]. Utilization of these methods may improve the accuracy of our method in the future study.

Nowadays, image-processing technologies are frequently combined with machine learning approaches, particularly supervised learning, such as the k-Nearest Neighbors [6,15,23], Support Vector Machine [11,25] and artificial neural network [4,14,40]. The accuracy of such approaches strongly depends on the training dataset. With regard to pixel-based segmentation in our method, as discussed by Guo *et al.* [26], training images must cover the full range of information of the targeted parts to be segmented so that accurate results can be obtained. The same can be said of blob-based segmentation. Furthermore, since the developed method requires two types of training datasets for both the pixel-based and blob-based segmentations, the preparation of the training images is a time-consuming task that may prove to be a bottleneck for the proposed method. Semi-automatic preparation of training data for blob-based segmentation using a *K*-means algorithm, which is used in related research [26,41], was also applied. However, this approach was observed to be inefficient for our method because different types of blobs were classified into the same clusters.

## 4. Conclusions

This paper proposes an image-processing method to accurately detect individual intact tomato fruits, including mature, immature and young fruits, on plant using a conventional RGB digital camera in conjunction with machine learning approaches. The proposed method consists of three steps. At the first step, pixel-based segmentation was conducted to roughly segment the pixels of the images into classes composed of fruits, leaves, stems and backgrounds. Blob-based segmentation was then conducted to eliminate misclassifications generated at the first step. At the third step, *X*-means clustering was applied to detect individual fruits in a fruit cluster. The developed method did not require an adjustment of the threshold values of each image for fruit detection because the image segmentations were conducted based on classification models generated by machine learning approaches. The results of fruit detection in the test images showed that the developed method achieved a recall of 0.80, while the precision was 0.88. In addition, the recall of young fruits was 0.78, although detection of young fruits is very difficult because of their small size and the similarity of their appearances with that of stems.

## Figures and Tables

**Table 1.** Results of fruit detection using the developed method.

	**Young**		**Immature**		**Mature**		**All Fruit**	

	Recall	*n*	Recall	*n*	Recall	*n*	Recall	Precision
All parts^†^	0.78	96	0.80	1323	1.00	35	0.80	0.88
Top^††^	0.85	96	0.84	282	-	-	0.82	0.77
Middle^††^	-	-	0.82	291	-	-	0.82	0.92
Bottom^††^	-	-	0.87	750	1.00	35	0.87	0.93

† Results of fruit detection using the model based on the training datasets of all parts;

†† Results of fruit detection using the model based on the training dataset prepared for each part individually.
